# MoDock: A multi-objective strategy improves the accuracy for molecular docking

**DOI:** 10.1186/s13015-015-0034-8

**Published:** 2015-02-18

**Authors:** Junfeng Gu, Xu Yang, Ling Kang, Jinying Wu, Xicheng Wang

**Affiliations:** State Key Laboratory of Structural Analysis for Industrial Equipment, Department of Engineering Mechanics, Dalian University of Technology, Dalian, 116023 China; Department of Computer Science and Technology, Dalian Neusoft Institute of Information, Dalian, 116023 China

**Keywords:** Multi-objective, Molecular docking, Scoring function, Optimization

## Abstract

**Background:**

As a main method of structure-based virtual screening, molecular docking is the most widely used in practice. However, the non-ideal efficacy of scoring functions is thought as the biggest barrier which hinders the improvement of the molecular docking method.

**Results:**

A new multi-objective strategy for molecular docking, named as MoDock, is presented to further improve the docking accuracy with available scoring functions. Instead of simple combination of multiple objectives with fixed weight factors, an aggregate function is adopted to approximate the real solution of the original multi-objective and multi-constraint problem, which will simultaneously smooth the energy surface of the combined scoring functions. Then, method of centers and genetic algorithm are used to find the optimal solution. Tests of MoDock against the GOLD test data set reveal the multi-objective strategy improves the docking accuracy over the individual scoring functions. Meanwhile, a 70% ratio of the good docking solutions with the RMSD value below 1.0 Å outperforms other 6 commonly used docking programs, even with a flexible receptor docking program included.

**Conclusions:**

The results show MoDock is an effective strategy to overcome the deviations brought by single scoring function, and improves the prediction power of molecular docking.

**Electronic supplementary material:**

The online version of this article (doi:10.1186/s13015-015-0034-8) contains supplementary material, which is available to authorized users.

## Background

Structure-Based Virtual Screening (SBVS) has become a routine tool in both pharmaceutical companies and academic groups for early-stage drug discovery [1]. As a main method of SBVS, molecular docking is the most widely used in practice, and there have reported a number of successful examples [2]. As a result, the docking method has received increasing interest in recent times. To date, over 60 docking programs and 30 scoring functions (SFs) have been disclosed [3]. For comparing their efficiency, there have been many comparative studies to evaluate the relative performance of the most popular programs and SFs [4-22]. However, previous comparative studies have revealed that none of the docking programs and SFs truly outperforms the others, and a universally accurate docking method is still out of reach.

It is fundamentally an optimization problem of docking a ligand into the binding site of a receptor. As the objects during the optimization process, SFs estimate binding affinities between small ligands and proteins, and rank the compounds, playing an essential role in molecular docking. The non-ideal efficacy of SFs is thought as the biggest barrier which hinders the improvement of the molecular docking method. The conflict between the accuracy and speed of SF is a difficult problem need to make great efforts in. More recently, many techniques have been applied to further improve the efficacy of SF, such as including thermodynamic data [23,24], including data derived from quantum chemical calculation [25,26], application of modern computation technique and computational intelligence [27,28], etc. Despite many achievements have been obtained, the development of an ideal SF still has a long way to go. Therefore, how to improve the docking accuracy with available SFs is a practical and urgent task. Most docking methods are based on one single objective, i.e., a SF. However, due to the approximation adopted in the SF developing, deviations from the real binding energy are unavoidable. Based on this consideration, consensus scoring was developed by combining multiple SFs to reduce the deviations brought by individual SFs as possible. The critical step in consensus scoring is the design of an appropriate consensus scoring strategy of individual scores so that the true modes/binders can be discriminated from others accordingly. However, classic consensus strategy like linear combination is strongly dependent on the initial parameters, and simple combination of multiple SFs will make the energy curves discontinuous and non-smooth, and make the optimization problem more difficult to solve.

The application of multiple SFs makes docking become a multi-objective optimization problem. How to choose and combine the SFs, and design relevant optimization strategy to the multi-objective problem are crucial for improving the docking efficiency with consensus scoring. In this work, a multi-objective docking strategy MoDock is proposed to further improve the pose prediction with available SFs. The SFs used in consensus scoring are preferred to be not correlated, so that errors can be diminished. The available scoring functions can generally be divided into the following three types: force-field-based, empirical-based and knowledge-based SFs. They focus on diverse aspects of ligand binding, and are derived from different principles. Therefore, three representative scoring functions from these three types are introduced as the objectives, and then a multi-objective optimization method is designed to optimize these three objectives simultaneously. The publicly available GOLD test set containing 134 protein-ligand complexes is applied to evaluate the reliability of MoDock. The results indicate that the multi-objective strategy can enhance the pose prediction power of docking with the available SFs.

## Models and Methods

### The optimization model and scoring functions

In this work, three representative SFs chosen from different categories are treated as objectives. Then mathematically, the docking problem can be written as follows:

1 $$ \begin{array}{l}\mathrm{M}\mathrm{i}\mathrm{n}\ \left\{{f}_1(X),{f}_2(X),{f}_3(X)\right\}\\ {}\mathrm{s}.\mathrm{t}.\ {g}_j(X)<0,j=1,2,\cdots q\end{array} $$

where *f*_*i*_(*X*) is the objective function derived from one of the three classes of SFs: the force-field-based, the empirical-based and the knowledge-based. *X* is the vector of design variables which serve to describe the conformational information of a ligand molecule. As we assume that the ligand is flexible and the receptor is rigid, *X* can then be expressed by the state variables of a molecule as follows:

2 $$ X={\left\{{T}_x,{T}_y,{T}_z,{R}_x,{R}_y,{R}_z,{T}_{b1},{T}_{b2},\cdots, {T}_{bn}\right\}}^T $$

where *T*_*x*_, *T*_*y*_, *T*_*z*_, *R*_*x*_, *R*_*y*_, *R*_*z*_ are the position coordinates and rotational angles of the entire ligand for the matching-based orientation search, and *T*_*b*1_, *T*_*b*2_, …, *T*_*bn*_ are the torsional angles of the rotatable bonds which account for the flexibility of the ligand.

The constraints *g*_*j*_(*X*), *j* = 1, 2, …, *q* is the size limits of the design variables shown as follows:

3 $$ \left\{\begin{array}{l}\underset{\bar{\mkern6mu}}{T_x}\ \le\ {T}_x\ \le\ \overline{T_x}\hfill \\ {}\underset{\bar{\mkern6mu}}{T_y}\ \le\ {T}_y\ \le\ \overline{T_y}\hfill \\ {}\underset{\bar{\mkern6mu}}{T_z}\ \le\ {T}_z\ \le\ \overline{T_z}\hfill \\ {}-\pi\ \le\ {R}_{x,y,z},\ {T}_{b1,\cdots, bn}\ \le\ \pi \hfill \end{array}\right. $$

The force-field-based scoring function adopts the classical AMBER molecular mechanics energy function [29,30], which approximates the binding affinity as the summation of van der Waals and electrostatic interactions, with an assumption that the hydrogen-bonding energies can largely be accounted for in the electrostatic term.

4 $$ {f}_1(X)={\displaystyle \sum_{i=1}^{n_{lig}}{\displaystyle \sum_{j=1}^{n_{rec}}\left(\frac{A_{ij}}{r_{ij}^{12}}-\frac{B_{ij}}{r_{ij}^6}+332.0\frac{q_i{q}_j}{D{r}_{ij}}\right)}} $$

where each term is a double sum over the ligand atom *i* and the receptor atom *j. n*_*lig*_, *n*_*rec*_ are the number of atoms in the ligand and the receptor, respectively; *A*_*ij*_, *B*_*ij*_ are van der Waals repulsion and attraction parameters, *r*_*ij*_ is the distance between atoms *i* and *j*, *q*_*i*_, *q*_*j*_ are the point charges on atoms *i* and *j*, *D* is dielectric function, and 332.0 is a factor that converts the electrostatic energy into kilocalories per mole.

As a commonly used empirical scoring function, X-Score is used here to evaluate the empirical-based scoring of the binding affinity [31]. It assumes that the van der Waals interaction (*E*_*vdw*_), hydrogen-bonding energy (*E*_*hb*_), hydrophobic (*E*_*hyd*_) and deformation (*E*_*def*_) terms are primary parts of binding energy. The scoring function is composed as follows with three ways for calculating the hydrophobic term.

5 $$ {f}_2(X)=\left(p{K}_{d,1}+p{K}_{d,2}+p{K}_{d,3}\right)/3 $$

6 $$ p{K}_{d,i}={C}_{0,i}+{C}_{1,i}{E}_{vdw}+{C}_{2,i}{E}_{hb}+{C}_{3,i}{E}_{def}+{C}_{4,i}{E}_{hyd,i} $$

The knowledge-based scoring function is more accurately referred to as Potential of Mean Force, PMF. Essentially, it is designed to reproduce the experimental structures rather than the binding energies. According to the inverse Boltzmann law, it can be directly derived from the statistical analysis of different types of atom pairs encoded in available crystal complex structures, and many approaches have emerged in recent years with different atom types and definitions of the reference state. We adopt the knowledge-based SF KScore in this work [32], which is defined as follow:

7 $$ {f}_3(X)={\displaystyle \sum_{\begin{array}{l} pl\\ {}r<{r}_{cut- off}\end{array}}{A}_{ij}(r)={\displaystyle \sum_{\begin{array}{l} pl\\ {}r<{r}_{cut- off}\end{array}}-{K}_BT \ln \left[{f}_{vol- corr}^j(r)\frac{\rho_{seg}^{ij}(r)}{\rho_{bulk}^{ij}}\right]}} $$

where *K*_*B*_ is the Boltzmann constant, *T* is the absolute temperature, ${f}_{vol- corr}^j$ is the ligand volume correction factor, ${\rho}_{seg}^{ij}(r)$ is the number density of atom pair *ij* that occurs in a spherical shell with a thickness of *Δr* ranging from *r* to *r* + *Δr*, and ${\rho}_{bulk}^{ij}$ expresses the number density when no interaction between *i* and *j* occurs.

### The multi-objective optimization strategy

Eq. (1) is a complex multi-objective and multi-constraint optimization problem, which is difficult to solve. Actually, there are more than twenty mathematical multi-objective optimization techniques, and most of them compromise the objects to find pareto-optimal solutions from which the optimal design is chosen for a certain application. In this work, method of centers is introduced to solve this multi-objective problem. This method introduces an upper bound *α*_*i*_ on each objective function and consecutively calculates the centers of intersection sets of the original feasible region with the level sets of objective functions. In initiation of the algorithm, all upper bounds *α*_*i*_ are set to be sufficiently large such that the iteration is forced to move towards the original feasible region, and then are reduced according to a certain criterion whenever the iterations become feasible

8 $$ {a}_i^k={r}_i{F}_i\left({X}^k\right) $$

where *k* is iteration number, and *r*_*i*_ is a weight parameter and is set to be 1.5 in this work. As such, the feasible region becomes smaller and smaller with iterations. *F*_*i*_(*X*^*k*^) is the normalized objective function, as three different score functions are adopted and their values cannot be directly compared. The normalized score *F*_*i*_(*X*^*k*^) are represented as follows:

9 $$ {F}_i\left({X}^k\right)=\frac{f_i\left({X}^k\right)}{f_i\left({X}^{k-1}\right)} $$

where *k* is the number of iteration in the optimizing process, and *X* is the optimal solution of the iteration.

Then, with the aggregate function proposed by *Li* [33], a smooth approximate function was constructed for transforming Eq. (1) to a single-objective and unconstraint problem

10 $$ \min\ S\left(X,\alpha \right)=\frac{1}{\rho } \ln {\displaystyle \sum_{i=1}^3 \exp \left[\rho \left({F}_i(X)-{\alpha}_i\right)\right]}+\frac{\lambda }{\rho } \ln {\displaystyle \sum_{j=1}^q \exp \left(\rho {g}_j(X)\right)} $$

where *ρ* is the coherent function parameter, and *λ* is the penalty factor. Theoretically, problem (10) and (1) will have the same solution when *ρ* → ∞. For numerical computation, *ρ* is set to be 10^5^ and *λ* = 100 in this work.

Despite the multi-objective docking problem (1) has been simplified with the method of centers and aggregate function, the objective function of problem (10) is still nonlinear and the design space is non-convex, which means we still need a powerful optimization tool to solve this problem. Genetic algorithms (GA) provide such a capability, and they have been successful adapted and implemented in a series of optimal design problems. In this work, an improved adaptive genetic algorithm is adopted [34], in which an entropy-based searching technique with multi-population is developed to ensure rapid and steady convergence. The GA firstly generates arbitrary *n* populations with the same design space, and the design space is treated as the initial searching space. Information entropy is used to measure the uncertainty which population the optimal solution occurs in. During the optimization process, the uncertainty and information entropy will be decreased. Meanwhile, the position of the optimal solution in the design space will be gradually clear, and the searching space will be narrowed until the optimal solution is found. The detailed steps of the genetic algorithm used in this study are given in the Additional file 1. In traditional GA, fixed genetic parameters such as the crossover and mutation probabilities *p*_*c*_ and *p*_*m*_ will lead to an unsteady and slow convergence of the optimization process. In the applied GA, *p*_*c*_ and *p*_*m*_ are treated as another two design variables which will also evolve in the execution of the applied GA. Therefore, these two parameters are self-adaptive, and rational determination of their values will be obtained. In this work, *p*_*c*_ and *p*_*m*_ are defined in [0.6, 1.0] and [0.0, 0.1], respectively.

### Preparation of the test data set

The main purpose of this study is to show the multi-objective strategy we proposed can improve the prediction accuracy with available popular SFs and the prediction accuracy are comparable with several popular docking programs. Therefore, the commonly used GOLD test data set, originally proposed by Jones et al. [35], was chosen for our studies. Each complex was separated into a probe molecule and a docking ligand according to the biological interacting pairs. Each protein molecule was obtained by excluding ligands, all structural water molecules, cofactors and metal ions from the receptor PDB file [36]. Next, a mol2 file was generated by adding hydrogen atoms and Kallman charge. Residues around the bound ligand within a radius of 6.5 Å were isolated from the protein to define as the active site. The ligands were then prepared by adding hydrogen atoms and Gasteiger-Marsili atomic charges. The heavy atoms number of the ligands ranged from 6 to 55, with 83.6% of the ligands possessing fewer than 30 such atoms. Besides, the rotatable bonds of the ligands ranged widely from 0 to 22, with greater than 88.8% of the ligands possessing fewer than 15 such bonds.

## Results and discussion

The evaluation of the docking accuracy is based on the root-mean-square deviation (RMSD) value of the locations of all heavy atoms in the model from those of the crystal structure. In general, the docking accuracy is acceptable if the RMSD value between the docked pose and X-ray crystal structure is less than 2.0 Å. Depending on the RMSD values, the accuracy is assigned to seven categories. The first, excellent, is for those predictions the top scoring pose of which is within 0.5 Å RMSD from the experimental results. The following three are for those good results with values between 0.5 and 2.0 Å. The fifth category, close, is used for those predictions the RMSD values of which are between 2.0 and 2.5 Å. The sixth category, error, is used for those predictions the RMSD values of which are between 2.5 and 3.0 Å. Finally, the seventh category, wrong, is used for completely incorrect predictions with RMSD values larger than 3.0 Å.

The docking RMSD results on the test set with the multi-objective strategy are listed in the Additional file 1.

### Multi-objective *versus* single-objective

The improvement of the multi-objective strategy against the single objectives is investigated first. Single-objective dockings with the above-mentioned three SFs separately are also performed on the test benchmark, and the RMSD values of the docking results are also listed in the Additional file 1.

**Figure 1:**
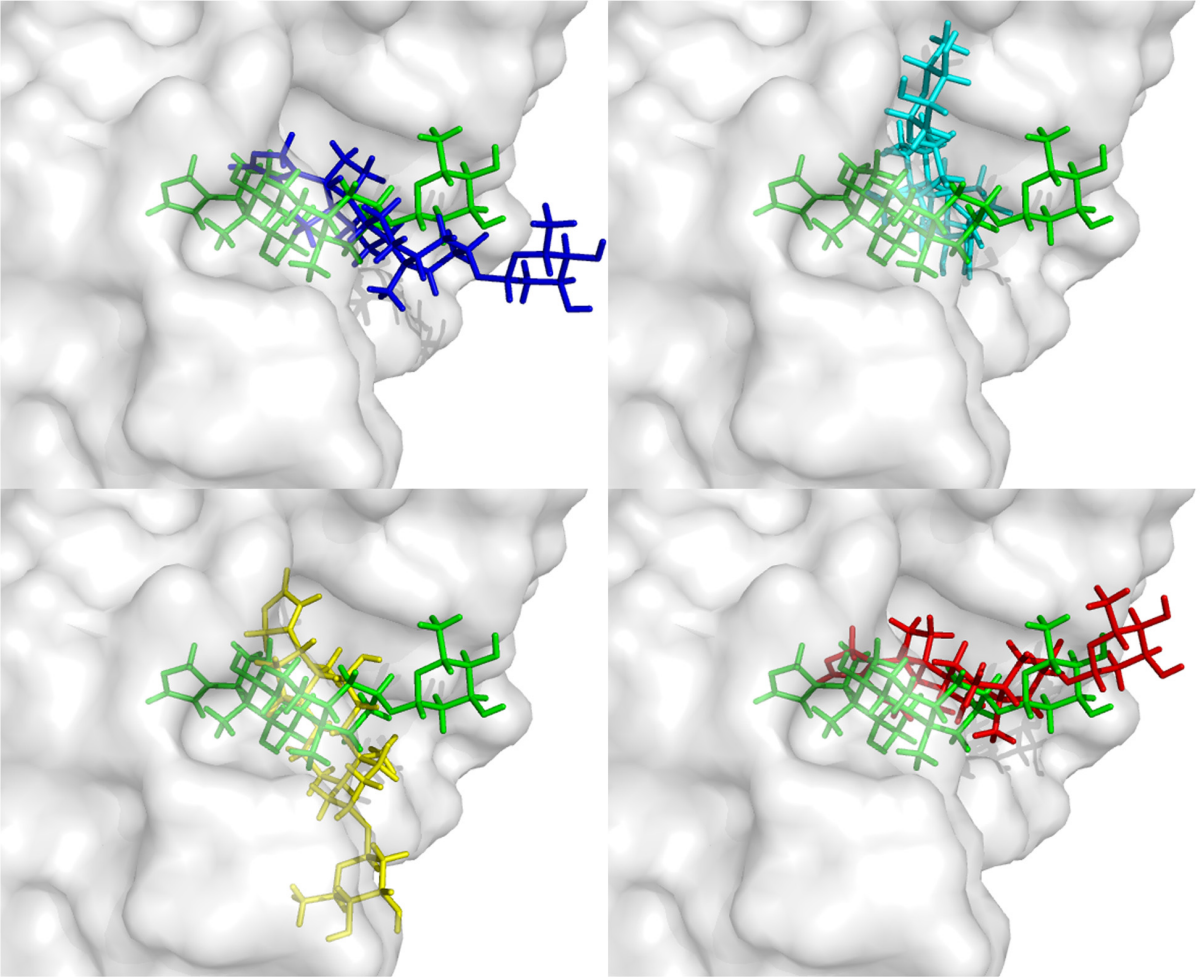
**The native and docked ligand poses of 1IGJ with different docking strategies.** The molecule shown in green is the native pose derived from the crystal structure; the pose docked with the multi-objective strategy is plotted in red, and the poses derived from single-objective docking are plotted in blue (force-field-based score), cyan (empirical-based score) and yellow (knowledge-based score).

PDB 1IGJ is an immunoglobulin complex with digoxin, and is chosen here to describe the docking procedure. The RMSD value of the multi-objective docking of 1IGJ is 0.83, and those of single-objective dockings with the force-field-based score, the empirical-based score and the knowledge-based score are 4.43, 1.01 and 6.13 respectively, and the docked poses are given in Figure 1. The molecule shown in green is the native pose derived from the crystal structure, and the binding score of the native pose is 16.06 (force-filed-based score), −9.64 (empirical-based score) and −120.74 (knowledge-based score). The molecules shown in blue, cyan and yellow are docked poses of single-objective dockings with the force-filed-based, empirical-based and knowledge-based score, and the binding scores are −29.37, −10.49, and −360.21 respectively. The score values show that the native pose does not correspond with the energy minima of any of these three scoring functions, so single-objective docking will not give a satisfactory result. The molecule shown in red is the docked pose with the multi-objective strategy, and the binding score is −12.54 (force-filed-based score), −9.75 (empirical-based score) and −138.84 (knowledge-based score), and the latter two are close to the score values of the native pose. From the view of this case, the multi-objective strategy will find a pose with more balanced energy distribution among the multiple SFs, thus decrease the deviations derived from individual SFs and find a more reasonable solution.

**Figure 2:**
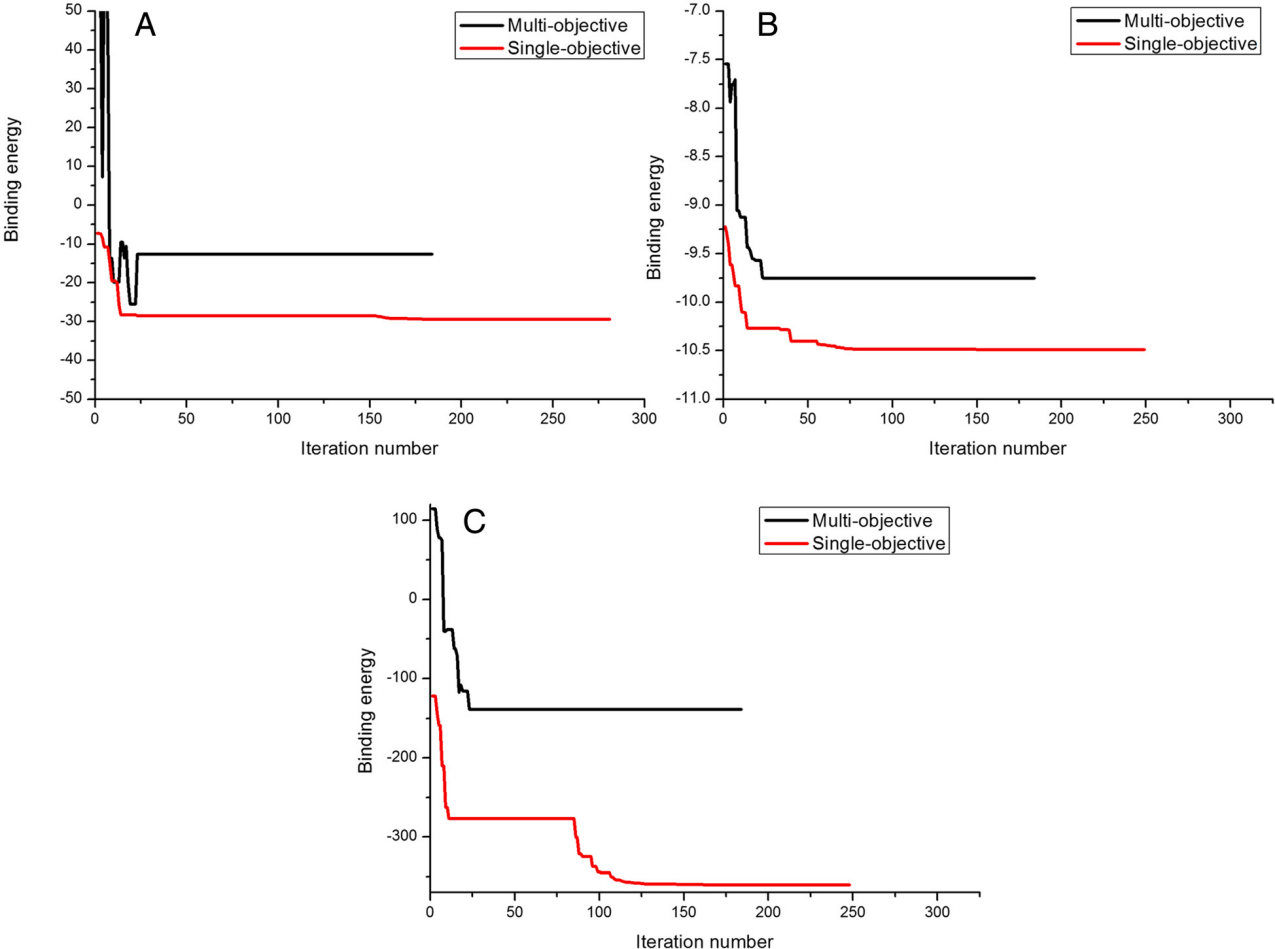
**The optimization procedures of 1IGJ with single-objective** ***versus*** **multi-objective strategy.** The multi-objective strategy is shown in black lines, and the single-objective strategy is shown in red lines: **(A)** with the force-field-based scoring function **(B)** with the empirical-based scoring function and **(C)** with the knowledge-based scoring function.

Figure 2 gives the docking optimization procedures with different scoring strategies. The black lines denote the change of SFs adopted in the multi-objective strategy, and the red lines are the SFs adopted in the single-objective dockings. In Figure 2A, the force-field-based score optimization procedures with the multi-objective and with the single-objective strategy are compared. The comparisons of the empirical-based score and knowledge-based score are plotted in Figure 2B and 2C respectively. The iteration number of the docking procedure with the multi-objective strategy is 184, and that of the single-objective docking with the force-field score is the longest 281, and the iteration numbers with the empirical score and knowledge-based score are 249 and 248 respectively. As indicated in the figure, the single-objective optimization is a monotonic decreasing procedure, while docking with the multi-objective strategy can hinder single SF to fall into its energy minimum.

**Figure 3:**
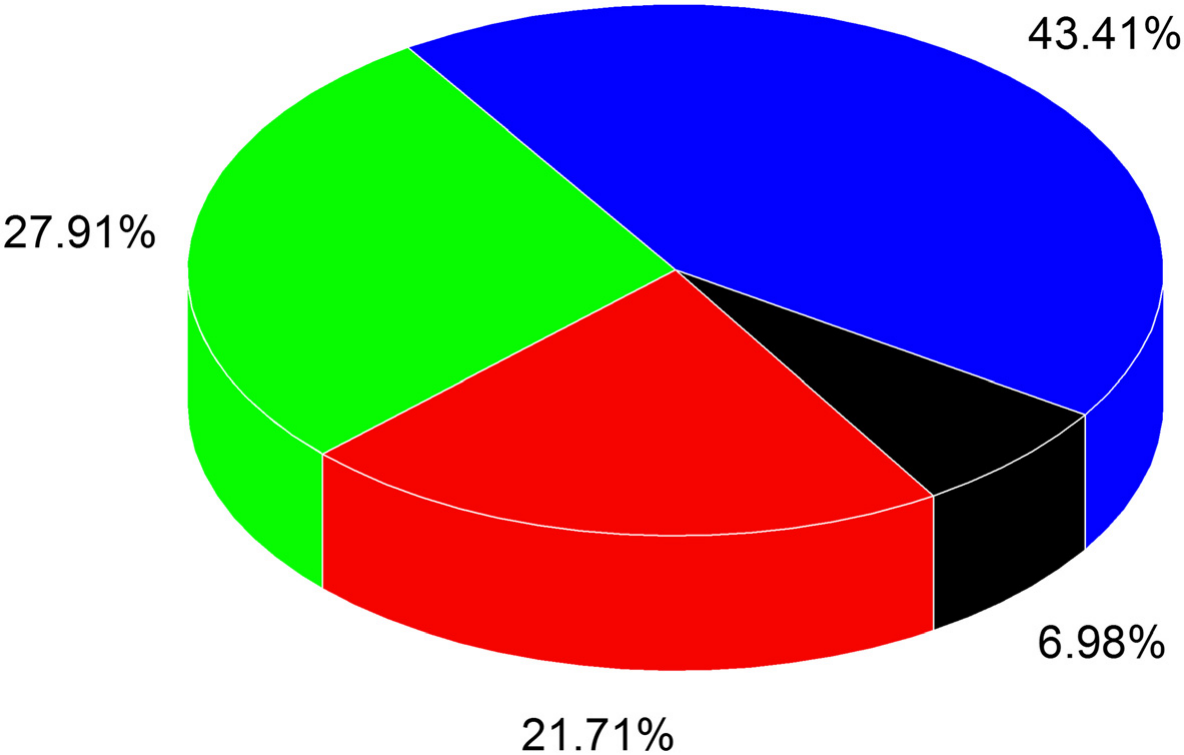
**The pie chart of comparison results between the multi-objective and single-objective docking strategy.** The colors denote the ratio of the multi-objective docking outperforms all (in blue), two (in green), one (in red) and none of the three single-objective dockings.

For a more comprehensive comparison of the docking accuracy with the multi-objective strategy over the single-objective strategy, a statistics on the docking RMSD values of 134 complexes is performed, and the statistical results are shown in Figure 3. As shown in the blue color, in 43.4% cases, the RMSD of the multi-objective docking is less than all those of the single-objective docking with all the three SFs. In 27.9% and 21.7% cases, the multi-objective docking outperforms two and one of the three single-objective dockings. Only in about 7.0% cases, the multi-objective strategy gets the worst results.

**Figure 4:**
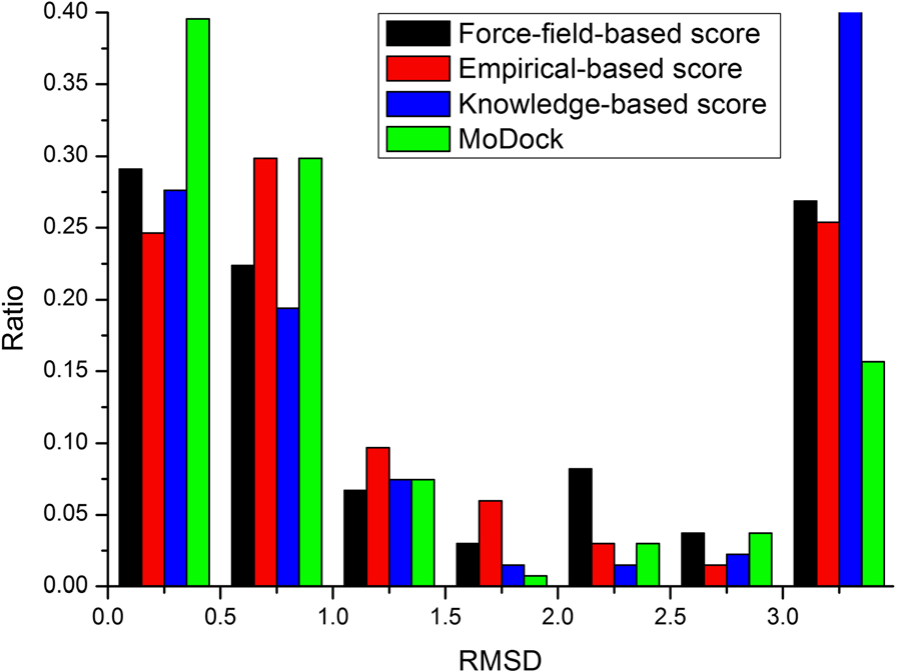
**Ratio comparison of docking accuracy between the multi-objective and single-objective docking strategy.**

A histogram is also plotted in Figure 4 to compare the docking accuracy of the multi-objective strategy versus the single-objective strategy in all the seven accuracy categories. As indicated in Figure 4, in the 0 ~ 0.5 and 0.5 ~ 1.0 intervals, the multi-objective strategy achieves the highest ratio, and the ratios are 39.6% and 29.9% respectively. Moreover, in the >3.0 interval, the multi-strategy achieves the lowest ratio 15.7%.

### Improvement over the other docking strategies

We also conduct a comparison of docking accuracy with other 5 commonly used docking programs: Glide [37], GOLD [35], Surflex [38], FlexX [39] and Dock6 [40]. These programs are performed with rigid receptor assumption. In addition, a flexible docking is performed with Dock6 separately with default parameters. Table 1 presents the ratios at different RMSD ranges of these programs. MoDock yields 53 (nearly 40%) excellent docking solutions with RMSD value below 0.5 Å, and 93 (nearly 70%) good predictions within 2.0 Å RMSD, which obviously outperforms the other programs which include the flexible receptor docking. In addition, there are 16% wrong predictions (RMSD value larger than 3.0 Å) with MoDock, which is the lowest among the rigid receptor docking programs and higher than the flexible receptor docking. Therefore, as shown in Table 1, the average RMSD of flexible docking have an almost equal value with our method, and far outperforms the other programs.

**Table 1:** **Docking accuracy comparison of MoDock with 6 commonly used docking programs**

**RMSD**	**Ratio**						
	**MoDock**	**Glide** ^**a**^	**GOLD** ^**b**^	**Surflex** ^**c**^	**FlexX** ^**d**^	**DOCK6** ^**e**^	**Dock6-F** ^**f**^
≤0.5	0.40	0.29	0.08	0.16	0.03	0.15	0.09
≥0.5, ≤1.0	0.30	0.19	0.27	0.32	0.18	0.15	0.32
≥1.0, ≤1.5	0.07	0.12	0.20	0.14	0.14	0.19	0.28
≥1.5, ≤2.0	0.01	0.11	0.11	0.15	0.14	0.13	0.11
≥2.0, ≤2.5	0.03	0.06	0.02	0.04	0.06	0.04	0.07
≥2.5, ≤3.0	0.04	0.03	0.03	0.02	0.04	0.08	0.03
≥3.0	0.16	0.20	0.28	0.17	0.40	0.27	0.09
Avg. RMSD	1.53	1.98	3.19	2.15	3.69	2.13	1.46

^a^the date are from reference [30].

^b^the date are from reference [29].

^c^the date are from reference [31].

^d^the date are from reference [32].

^e^the date are from reference [28].

^f^the date are derived from running Dock6 with default parameters.

From this point of view, flexible docking can decrease the ratio of wrong prediction by considering the conformational change of the receptor during the receptor-ligand binding process. However, the flexible docking method is also seriously limited by the accuracy of the score function it used, so the ratio of excellent docking cannot be increased.

### Failure analysis

**Figure 5:**
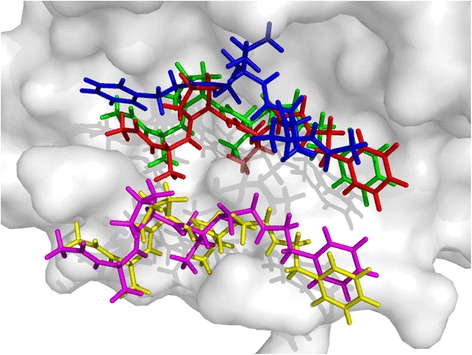
**The native and docked ligand poses of 4EST with different docking strategies.** The molecule shown in red is the native pose derived from the crystal structure; the pose docked with MoDock is plotted in magentas, and the poses derived from single-objective docking are plotted in blue (force-filed-based score), green (empirical-based score) and yellow (knowledge-based score).

As shown in Table S1 Additional file 1, there are 21 (about 16%) wrong predictions with MoDock. In most of the failure cases, none of the three scoring functions can get a good result with single-objective strategy, which means they seriously deviate from the real energy distribution. The poses of their energy minima are far away from the native pose, and they probably have high energy barriers at the native pose. In the circumstances, the multi-objective strategy can rarely find a good docking solution. While in other cases, one or two of the three scoring functions get good docking results, and the multi-objective strategy still cannot find a reasonable solution. For explaining the reason, we choose a case 4EST for a further analysis. The native and docked poses are overlapped and plotted in Figure 5. The native pose are plotted in red, and the binding energy is 8310.42 (force-filed-based score), −8.77 (empirical-based score) and −173.45 (knowledge-based score). The pose docked with empirical-based score is plotted in green, which has the best docking accuracy 0.52 Å, and the binding energy is 3426.94 (force-filed-based score), −9.04 (empirical-based score) and −166.90 (knowledge-based score). The pose docked with force-field-based score is plotted in blue, which has the worst docking accuracy and even the orientation is completely opposite to the native pose, and the binding energy is −18.93 (force-field-based score), −6.42 (empirical-based score) and 239.36 (knowledge-based score). The pose docked with knowledge-based score is plotted in yellow, and the binding energy is 3203.61 (force-field-based score), −6.89 (empirical-based score) and −219.73 (knowledge-based score). The pose docked with MoDock is plotted in magentas, and the binding energy is −13.22 (force-field-based score), −6.49 (empirical-based score) and −118.91 (knowledge-based score). From the poses in Figure 5 and their energy distribution, docking with single-objective can find lower energy pose, but whether the pose is close to the native pose depends on whether the adopted scoring function has an energy minimum near the native pose. In this case, the empirical-based score has an energy minimum near the native pose, so docking with empirical score finds an excellent pose with RMSD value under 1.0 Å. However, the force-field-based score has a very high binding energy on and near the native pose, and the optimization on the force-field-based score results in the change of the orientation of the ligand. The multi-objective strategy will consider all the three SFs during the docking process, and it will find a balanced pose among these three scoring functions. Therefore, the abnormal energy distribution of the force-field-based score is the reason of the failure of the docking with MoDock.

## Conclusions

In this work, we present a new multi-objective strategy MoDock for improving the accuracy of the molecular docking. To reduce the correlations between each other, three scoring functions chosen from different categories—force-field-based, empirical-based and knowledge-based, are treated as the objectives during the docking optimization. Instead of simple combination with predefined weight factors, an aggregate function is adopted to combine the multiple objectives and to approximate the real solution of the original multi-objective and multi-constraint problem. Finally, method of centers and genetic algorithm are introduced to solve the optimization problem.

The results of the docking experiments on the 134 diverse complexes from the GOLD test data set have shown an obvious improvement of the docking accuracy. Detailed analysis shows that in about half of the cases, the multi-objective docking strategy outperforms all the single-objective dockings. In addition, MoDock yielded 93 (nearly 70%) good docking solutions with a RMSD value below 1.0 Å, which clearly outperforms 5 rigid receptor and 1 flexible receptor docking programs, and the average RMSD value is only slightly higher than the flexible receptor docking program. The results indicate that the multi-objective strategy can overcome the deviations brought by single scoring function, so it makes the strategy an effective method to improve the docking accuracy with available scoring functions. However, through the analysis of the failure cases, we find the multi-objective strategy still limited by the combined SFs. If the energy distribution of a scoring function is seriously deviated, then it will result in the failure of the multi-objective strategy. Therefore, continuous development of the docking scoring function will further improve the accuracy of the multi-objective strategy.

In this work, we only consider three diverse scoring functions—AMBER, X-Score and KScore to demonstrate the efficacy of the multi-objective strategy, but the strategy is not restricted to these scoring function and even not to the quantity of the scoring functions. Different scoring functions can be combined with this multi-objective strategy to research the best combinations, and this will be the direction of our next work.

## Additional file

Additional file 1:
**The RMSD results of dockings on the 134 complexes of GOLD test set with different docking strategy; the detailed steps of the genetic algorithm used in this study.**
