# Supplementary material for: MoDock: A multi-objective strategy improves the accuracy for molecular docking
Source: Algorithms Mol Biol. 2015 Feb 18;10:8. doi: 10.1186/s13015-015-0034-8 (PMC4336518; doi:10.1186/s13015-015-0034-8)
Supplement: Additional file 1: — The RMSD results of dockings on the 134 complexes of GOLD test set with different docking strategy; the detailed steps of the genetic algorithm used in this study. [file 13015_2015_34_MOESM1_ESM.doc]

**Table S1 The RMSD results of dockings on the 134 complexes of GOLD test set with different docking strategies**

| PDB code | MoDock | Single-objective | | |
| --- | --- | --- | --- | --- |
|  |  | Force-field-based | Empirical-based | Knowledge-based |
| 1aaq | 0.6 | 0.55 | 0.53 | 0.45 |
| 1abe | 0.85 | 0.23 | 0.74 | 0.77 |
| 1acj | 1.29 | 0.37 | 2.26 | 4.92 |
| 1ack | 0.83 | 4.43 | 1.01 | 6.13 |
| 1acl | 1.37 | 0.54 | 2.48 | 7.56 |
| 1acm | 1.64 | 0.84 | 1.86 | 3.86 |
| 1aco | 1.44 | 0.32 | 1.46 | 0.23 |
| 1aec | 6.52 | 6.71 | 0.86 | 0.55 |
| 1aha | 0.32 | 0.29 | 0.39 | 0.26 |
| 1apt | 0.29 | 8.4 | 0.41 | 0.34 |
| 1ase | 2.49 | 3.09 | 4.99 | 1.01 |
| 1atl | 1.14 | 6.05 | 1.79 | 0.72 |
| 1azm | 0.8 | 0.3 | 0.89 | 5.57 |
| 1baf | 0.32 | 1.61 | 9.11 | 8.87 |
| 1bbp | 0.56 | 0.45 | 0.76 | 0.17 |
| 1blh | 3.33 | 2.17 | 4.34 | 3.11 |
| 1bma | 0.62 | 2.75 | 0.4 | 0.42 |
| 1byb | 0.36 | 0.75 | 0.44 | 0.39 |
| 1cbs | 0.42 | 1.11 | 5.45 | 0.62 |
| 1cbx | 0.48 | 0.53 | 0.71 | 1.03 |
| 1cdg | 0.58 | 0.97 | 0.71 | 2.41 |
| 1cil | 0.44 | 0.45 | 2.1 | 4.29 |
| 1com | 5.31 | 0.87 | 4.9 | 4.15 |
| 1coy | 0.24 | 0.4 | 1.65 | 5.16 |
| 1cps | 0.88 | 0.27 | 0.96 | 1.21 |
| 1ctr | 1.31 | 3.53 | 3.27 | 12.42 |
| 1dbb | 0.42 | 1.24 | 0.97 | 0.93 |
| 1dbj | 0.7 | 0.39 | 0.87 | 0.65 |
| 1did | 0.17 | 0.38 | 0.85 | 0.25 |
| 1die | 0.83 | 2.41 | 0.27 | 0.93 |
| 1dr1 | 0.75 | 0.45 | 1.06 | 4.52 |
| 1dwd | 0.47 | 0.59 | 0.46 | 4.12 |
| 1eap | 0.47 | 5.64 | 1.03 | 0.35 |
| 1eed | 11.11 | 14.44 | 6.35 | 5.27 |
| 1epb | 0.49 | 0.51 | 0.63 | 1.93 |
| 1etr | 4.42 | 10.59 | 2.77 | 2.51 |
| 1etr | 0.3 | 8.88 | 0.42 | 0.42 |
| 1fen | 0.39 | 0.6 | 0.42 | 3.55 |
| 1fkg | 0.52 | 0.23 | 5.75 | 0.69 |
| 1fki | 0.63 | 2.84 | 0.83 | 1.06 |
| 1frp | 0.61 | 0.16 | 1.19 | 0.35 |
| 1ghb | 0.67 | 0.71 | 4.05 | 4.05 |
| 1glp | 0.72 | 0.73 | 0.46 | 6.99 |
| 1glq | 2.29 | 5.67 | 0.37 | 0.36 |
| 1hdc | 0.26 | 0.14 | 8.07 | 7.83 |
| 1hdy | 0.68 | 2.88 | 0.6 | 3.12 |
| 1hef | 5.55 | 5.02 | 11.43 | 7.55 |
| 1hfc | 0.35 | 6.98 | 6.88 | 0.58 |
| 1hri | 0.5 | 0.36 | 0.62 | 0.65 |
| 1hsl | 0.2 | 0.36 | 0.3 | 0.23 |
| 1hyt | 0.45 | 0.47 | 0.41 | 0.56 |
| 1icn | 0.7 | 0.74 | 1.32 | 8.88 |
| 1ida | 0.41 | 0.22 | 0.44 | 0.34 |
| 1igj | 0.68 | 3.19 | 7.26 | 7.89 |
| 1imb | 2.92 | 2.92 | 3.82 | 3.05 |
| 1ive | 4.6 | 3.8 | 6.34 | 4.44 |
| 1lah | 0.19 | 0.21 | 0.19 | 0.2 |
| 1lcp | 0.52 | 2.4 | 1.83 | 4.67 |
| 1ldm | 4.03 | 1.81 | 5.08 | 0.65 |
| 1lic | 0.64 | 0.64 | 1.37 | 10.96 |
| 1lmo | 0.98 | 7.2 | 2.33 | 4.04 |
| 1lna | 0.3 | 0.66 | 0.54 | 5.15 |
| 1lpm | 3 | 6.55 | 1.61 | 0.82 |
| 1lst | 0.15 | 0.18 | 1.61 | 0.21 |
| 1mcr | 3.27 | 2.3 | 3.97 | 4.38 |
| 1mdr | 1.23 | 0.61 | 1.23 | 0.35 |
| 1mmq | 0.5 | 0.31 | 6.15 | 1.56 |
| 1mrg | 0.8 | 0.53 | 0.81 | 0.41 |
| 1mrk | 0.55 | 0.84 | 1.59 | 1.41 |
| 1mup | 1.24 | 0.57 | 3.39 | 5.12 |
| 1nco | 0.5 | 11.9 | 0.46 | 0.33 |
| 1nis | 0.34 | 0.23 | 0.34 | 1.18 |
| 1pbd | 0.26 | 0.26 | 0.28 | 0.27 |
| 1pha | 0.46 | 0.46 | 1.01 | 4.46 |
| 1phd | 6.84 | 5.45 | 8.48 | 9.53 |
| 1phg | 0.33 | 3.36 | 9.33 | 9.25 |
| 1poc | 0.36 | 11.33 | 0.8 | 0.42 |
| 1rds | 0.37 | 3.75 | 0.49 | 0.36 |
| 1rne | 13.2 | 14.49 | 0.7 | 13.37 |
| 1rob | 0.58 | 0.9 | 0.64 | 0.86 |
| 1slt | 1.12 | 1.01 | 5.95 | 2.07 |
| 1snc | 0.71 | 0.45 | 3.67 | 1.15 |
| 1srj | 0.99 | 2.44 | 0.73 | 0.76 |
| 1stp | 0.3 | 7.42 | 0.45 | 0.49 |
| 1tdb | 5.4 | 0.99 | 8.83 | 8.5 |
| 1tka | 4.32 | 8.68 | 5.63 | 5.48 |
| 1tmn | 0.53 | 7.41 | 0.69 | 6.01 |
| 1tng | 0.38 | 0.34 | 0.72 | 0.97 |
| 1tni | 4.94 | 1.72 | 0.87 | 2.96 |
| 1tnl | 0.14 | 2.27 | 0.37 | 0.16 |
| 1tph | 5.3 | 0.24 | 5.19 | 0.3 |
| 1tpp | 3.36 | 2.11 | 1.05 | 7.18 |
| 1trk | 6.67 | 8.72 | 5.95 | 8.23 |
| 1tyl | 2.53 | 2.53 | 3.53 | 4.04 |
| 1ukz | 0.15 | 0.42 | 0.28 | 4.89 |
| 1ulb | 0.65 | 3.09 | 1 | 3.89 |
| 1wap | 0.28 | 0.26 | 0.27 | 0.4 |
| 1xid | 2.45 | 2.09 | 2.55 | 3.42 |
| 1xie | 0.24 | 2.36 | 4.6 | 4.62 |
| 2ada | 0.17 | 0.31 | 0.24 | 0.27 |
| 2ak3 | 0.31 | 3.18 | 0.42 | 0.56 |
| 2cgr | 0.81 | 0.71 | 0.81 | 0.55 |
| 2cht | 0.95 | 1.17 | 0.74 | 0.38 |
| 2cmd | 0.28 | 0.63 | 0.35 | 0.36 |
| 2ctc | 0.44 | 0.49 | 0.62 | 0.96 |
| 2dbl | 0.4 | 3.15 | 0.24 | 0.69 |
| 2gbp | 0.28 | 0.34 | 0.44 | 0.11 |
| 2lgs | 4.71 | 1.21 | 1.41 | 4.53 |
| 2mcp | 0.49 | 2.46 | 0.93 | 1.03 |
| 2mth | 5.21 | 5.35 | 4.37 | 5.87 |
| 2phh | 0.54 | 3.04 | 0.64 | 0.44 |
| 2pk4 | 2.44 | 1.27 | 0.82 | 1.32 |
| 2plv | 0.68 | 0.68 | 0.99 | 0.84 |
| 2r07 | 0.47 | 1.33 | 0.47 | 0.95 |
| 2sim | 0.66 | 4.15 | 0.77 | 1.3 |
| 2yhx | 2.61 | 2.15 | 7.18 | 6.61 |
| 3aah | 0.32 | 0.39 | 0.35 | 0.48 |
| 3cla | 1.26 | 4.63 | 1.38 | 5.48 |
| 3cpa | 0.71 | 0.51 | 0.76 | 0.6 |
| 3gch | 2.69 | 5.11 | 3.06 | 2.6 |
| 3hvt | 0.54 | 0.9 | 0.74 | 0.4 |
| 3ptb | 0.35 | 1.52 | 0.26 | 0.3 |
| 3pti | 0.26 | 0.3 | 0.27 | 0.37 |
| 4cts | 3.23 | 0.66 | 4.04 | 3.13 |
| 4dfr | 0.43 | 0.48 | 0.51 | 9.4 |
| 4est | 9.04 | 11.11 | 0.52 | 8.79 |
| 4fab | 0.95 | 0.82 | 1.97 | 5.29 |
| 4phv | 0.45 | 0.32 | 11.54 | 6.02 |
| 5p2p | 0.24 | 0.28 | 0.53 | 0.49 |
| 6abp | 0.84 | 0.39 | 0.71 | 0.73 |
| 6rnt | 0.43 | 1.07 | 0.71 | 6.11 |
| 6rsa | 0.75 | 0.89 | 0.43 | 0.78 |
| 7tim | 1.2 | 0.69 | 1.1 | 0.6 |
| 8gch | 0.53 | 1.34 | 0.44 | 0.45 |

**The detailed steps of the genetic algorithm used in this study**

**Step 1.** Set the convergence tolerance *ƞ*=10^-3^, iteration number *k*=0, upper bounds $\alpha_{i}^{0}$=10^6^, where *i*=1, 2, 3 is the index of the objective.

**Step 2.** Give the global initial searching space *D*_0_(0), set the evolution generation of the genetic algorithm *l*=0, and generate arbitrary *n*=6 initial populations composed of *m*=30 members.

**Step 3.** For every individual in *n* populations:

**Step 3.1.** Calculate the force-field-based score based on Eq. (4).

**Step 3.2.** Calculate the empirical-based score based on Eq. (5) and (6).

**Step 3.3.** Calculate the knowledge-based score based on Eq. (7).

**Step 3.4.** Calculate the approximate function *S*(*X*, *α*) based on Eq. (10).

**Step 4.** Calculate the fitness function based on the approximate function for every individual, and then perform reproduction, crossover and mutation.

**Step 5.** In each population, sort the individuals based on their fitness scores. Then, sort the best individuals of all the populations, and record the population *pop* of which the best individual has the worst fitness score. Calculate the probability *p_j_* that the optimal solution occurs in the population *j* and the narrowing coefficients of the searching space *R_j_*.

**Step 6.** Calculate the narrowed searching space *D_j_*(*l*+1), and modify the upper and lower bounds of variables accordingly. For population *pop*, change its searching space back to the initial searching space.

**Step 7.** Generate *n* populations on the narrowed searching space *D_j_*(*l*+1).

**Step 7.** *l*=*l*+1, Go to **step 3** until the convergence criterion *D_j_*(*l*+1)≤*ε* is satisfied, where *ε*=10^-6^ is a given tolerance.

**Step 8.** Output the optimization results *S^k^* and the corresponding *X^k^*. *k*=*k*+1, update $\alpha_{i}^{k}$ based on Eq. (8), and go to **step 2** until the convergence criterion $\left|S^{k}-S^{k-1}\right|\leq\eta$ is satisfied.

**Step 9.** Output the optimization results, and stop the iteration process.
